# Student perceptions of artificial intelligence in higher education: a structural analysis at an Italian university

**DOI:** 10.3389/fpsyg.2026.1861001

**Published:** 2026-06-29

**Authors:** Calogero Cammà, Luisa Amenta, Salvatore Battaglia, Ciro Celsa, Giansalvo Cirrincione, Salvatore Contino, Pier Paolo Corso, Salvatore Di Dio, Gabriele Di Maria, Marco Enea, Adriano Fagiolini, Alessandra Ferraro, Mauro Giuffrè, Giosuè Lo Bosco, Domenica Matranga, Roberto Pirrone, Francesco Maria Raimondi, Ilenia Tinnirello, Riccardo Uccello, Marco Vaccaro, Caterina Ventimiglia, Salvatore Vitabile, Massimo Midiri

**Affiliations:** 1 Department of Health Promotion, Mother & Child Care, Internal Medicine & Medical Specialties, Gastroenterology & Hepatology Unit, University of Palermo, Palermo, Italy; 2 Dipartimento di Scienze umanistiche, University of Palermo, Palermo, Italy; 3 Department of Economics Business and Statistics, University of Palermo, Palermo, Italy; 4 Département Electronique-Electrotechnique-Automatique (EEA), University of Picardie Jules Verne, Amiens, France; 5 Department of Engineering, University of Palermo, Palermo, Italy; 6 Dipartimento di Fisica e Chimica-Emilio Segré, University of Palermo, Palermo, Italy; 7 Dipartimento di Architettura, University of Palermo, Palermo, Italy; 8 Department of Health Promotion, Mother and Child Care, Internal Medicine, and Medical Specialties (PROMISE), University of Palermo, Palermo, Italy; 9 Department of Engineering, Mobile & Intelligent Robots @ Panormus Laboratory (MIRPALab), University of Palermo, Palermo, Italy; 10 University of Palermo, Palermo, Italy; 11 Department of Internal Medicine (Digestive Diseases), Yale School of Medicine, New Haven, CT, United States; 12 Department of Medical, Surgical and Health Sciences, University of Trieste, Trieste, Italy; 13 Department of Mathematics and Computer Science, University of Palermo, Palermo, Italy; 14 University Information Systems Area, University of Palermo, Palermo, Italy; 15 Department of Law, University of Palermo, Palermo, Italy; 16 Department of Biomedicine, Neurosciences and Advanced Diagnostics (BIND), University of Palermo, Palermo, Italy

**Keywords:** artificial intelligence, confirmatory factor analysis, educational technology, factor analysis, higher education, student perceptions

## Abstract

**Background and aims:**

The rapid advancement of artificial intelligence (AI) is transforming higher education, yet understanding of student perceptions remains limited. This study investigates the structure of student attitudes toward AI and identifies key predictors among Italian university students.

**Methods:**

A cross-sectional survey was administered to 864 students at the University of Palermo (May–June 2025). The questionnaire examined demographics, AI experience, and attitudes using 10 Likert-type items. Data were analyzed using exploratory factor analysis and confirmatory factor analysis.

**Results:**

Most students (65.8%) reported superficial AI knowledge, yet 93.6% had used AI applications, predominantly ChatGPT (59.8%). Factor analyses identified two distinct latent constructs: perceived Impact of AI and AI-related Concerns, negatively correlated (*r* = −0.34, *p* < 0.001). Perceived Impact was positively predicted by age (β = 0.17), STEM (β = 0.19), Health/Agricultural/Veterinary sciences (β = 0.23), Economics/Law/Social Sciences (β = 0.16), and regular AI use, while negatively predicted by female gender (β = −0.09) and non-use (β = −0.35). AI-related Concerns were positively predicted by female gender (β = 0.21) and non-regular use (never: β = 0.20; occasionally: β = 0.29), and negatively by STEM (β = −0.11) and Health sciences (β = −0.15).

**Conclusions:**

Student attitudes toward AI reflect two distinct dimensions: opportunity recognition and risk awareness, systematically influenced by gender, discipline, and AI experience. Successful implementation requires tailored approaches addressing gender-specific concerns, discipline-specific needs, and promoting direct AI experience.

## Introduction

The integration of artificial intelligence (AI) into higher education represents one of the most significant technological shifts in contemporary academic environments (Chen et al., 2020; Zawacki-Richter et al., 2019). As AI technologies become increasingly sophisticated and accessible, universities worldwide are addressing complex challenges regarding how, when, and to what extent these tools should be incorporated into educational curricula and learning practices (Holmes et al., 2019).

Recent developments in generative AI, particularly large language models like ChatGPT, have accelerated discussions about AI's role in education (Kelly and Sullivan, 2023). These technologies offer unprecedented opportunities for personalized learning, automated assessment, and research acceleration, while simultaneously raising concerns about academic integrity, critical thinking skills, and the fundamental nature of human learning (Rudolph et al., 2023).

Despite the growing interest in educational AI applications, there remains a significant gap in understanding how students—as primary beneficiaries and end-users of these technologies—perceive AI integration into their academic programs (Popenici and Kerr, 2017). While previous studies have examined general attitudes toward educational technology, few have systematically investigated the underlying dimensional structure of student perceptions regarding AI in higher education (Akgun and Greenhow, 2022). Understanding whether student attitudes represent a unidimensional construct or reflect multiple independent dimensions (such as opportunity recognition vs. risk awareness) is crucial for developing targeted implementation strategies.

Moreover, existing research has largely focused on descriptive assessments of attitudes without examining how demographic characteristics, academic discipline, and prior AI experience systematically influence these perceptions. Identifying predictors of positive and negative attitudes can inform evidence-based approaches to AI literacy programs and help address barriers to adoption among specific student subgroups.

Student perceptions of AI vary considerably across cultural and institutional contexts. In Asian higher education settings, research conducted in Chinese university environments has documented that students simultaneously recognize AI's potential for enhancing efficiency while expressing persistent concerns about content accuracy, over-reliance, and ethical implications (Tian et al., 2024). In the Middle East, studies have shown that institutional readiness, digital infrastructure, and cultural norms significantly shape AI adoption patterns among university students (Alshammari et al., 2025). Within Western countries, emerging research has examined student attitudes toward AI across multiple national contexts: studies conducted across multiple European countries (Schei et al., 2024) and Latin America (Gutiérrez-Leefmans et al., 2026) have highlighted that national educational policies and institutional digital readiness meaningfully modulate AI adoption patterns, with students expressing both enthusiasm for AI's learning potential and apprehension about ethical risks, although with notable cross-national variation. Against this backdrop, the Italian higher education context remains underexplored, and it is unclear whether findings from other national settings extend to the Italian case.

The Italian higher education context presents a particularly interesting case study, as it combines traditional academic structures with increasing pressure for digital transformation. Italian universities are characterized by diverse disciplinary traditions, from humanities and social sciences to science, technology, engineering and mathematics (STEM) and health sciences, each of which may approach AI integration differently. Understanding how student perceptions vary across these disciplines can provide valuable insights for policy makers and educational leaders managing AI integration decisions in similar contexts.

This study addresses these knowledge gaps by conducting a comprehensive survey at the University of Palermo (Italy), examining student perceptions of AI technologies through a confirmatory factor analysis approach. Specifically, we aim to: (1) identify the key dimensions that characterize student attitudes toward AI integration in higher education; (2) examine the relationships between these attitude dimensions; and (3) determine how demographic characteristics, academic discipline, and AI usage patterns predict these underlying constructs.

## Materials and methods

### Study design and setting

This cross-sectional survey study was conducted at the University of Palermo (UNIPA), Italy, between May and June 2025. The University of Palermo is a comprehensive public research university with approximately 46,000 students across more than 130 degree programs, spanning 16 different academic departments and disciplines. The response rate was 1.88% (864 completed surveys out of approximately 46,000 eligible students).

### Participants

The study targeted all enrolled students across three educational levels: (1) undergraduate students in 3-year degree programs; (2) graduate students in master's degree and single-cycle master's degree programs; and (3) doctoral students. The total sample comprised 864 students representing diverse academic areas including STEM, Medicine and Life Sciences, Economics and Law, and Arts, Literature and Educational Sciences.

### Survey instrument

A comprehensive questionnaire was developed covering the following domains:

1. **Demographic Information**: age, gender, degree type, year of study, and academic area/field of study.
2. **AI Knowledge and Experience**: self-assessed level of AI knowledge, current usage frequency of AI applications, and specific AI tools and platforms used.
3. **Attitudes And Perceptions**: A Set of 10 Likert-type items designed to measure student perceptions of AI integration in higher education. These items assessed: (a) perceived impact of AI on professional field, quality of university education, importance of AI competencies, support for mandatory AI courses, and interest in deepening AI knowledge (Items 1–5); and (b) concerns about reduced human interaction in learning, increased digital divide, privacy and data security risks, excessive technological dependency, and impact on future employment (Items 6–10). Responses were recorded on a 5-point Likert scale. The 10 Likert-type items measuring student attitudes toward AI were developed *de novo*, as existing validated instruments—including the SATAI (Suh and Ahn, 2022), the AIAS-4 (Grassini, 2023), and the GAAIS (Schepman and Rodway, 2023)—were not designed to capture the multidimensional structure of student perceptions in higher education contexts. Item development followed a co-creation process involving a representative panel of faculty members and students. Prior to data collection, a panel of experts in educational pedagogy reviewed the items for content validity and relevance, followed by a group of researchers not involved in the study who assessed comprehensibility and identified ambiguous or redundant items. Given the absence of an established theoretical model as starting point, the questionnaire is exploratory in nature; the factor structure identified through the subsequent analyses is intended as a preliminary framework for future instrument refinement and validation.
4. **Preferred integration methods and priorities**: including preferred delivery formats (mandatory courses, optional courses, integrated modules, seminars) and priority areas for AI education (practical applications, tool usage, theoretical foundations, ethical implications).
5. **Open-ended questions**: about opportunities for AI integration, perceived challenges, and obstacles.

The full text of the questionnaire is reported in Supplementary material.

### Data collection

Surveys were distributed via institutional email to all eligible students. The survey was administered using open-source LimeSurvey and remained open for 4 weeks. Reminder emails were sent at regular intervals to maximize response rates.

### Data analysis

Quantitative data were analyzed using descriptive statistics, including frequencies, percentages, means, and standard deviations for demographic characteristics, AI knowledge levels, usage patterns, and preferences for integration methods.

### Exploratory factor analysis (EFA)

To investigate the underlying structure of student attitudes toward AI, we performed an exploratory factor analysis (EFA) on the 10 Likert-type items designed to measure perceptions of AI integration. The correlation matrix was inspected, and sampling adequacy was verified through standard criteria. Factor extraction relied on maximum likelihood estimation, which allows for inferential testing and the computation of fit indices under the assumption of multivariate normality. The number of factors was determined by visual inspection of the scree plot and parallel analysis, a procedure shown to outperform traditional eigenvalue >1 criteria.

Both orthogonal (varimax) and oblique (oblimin) rotations were examined to assess factor interpretability. Internal consistency of each factor was evaluated with Cronbach's alpha and McDonald's omega.

### Confirmatory factor analysis (CFA)

Based on the EFA results, a confirmatory factor model was specified with items loading on their respective latent factors. Latent factors were allowed to correlate, and a theoretically motivated residual covariance was included between Item 1 and Item 10. This decision was justified as follows: Item 1 (“AI will have a significant impact on my future professional field”) measures the perceived extent to which AI will transform one's occupational domain, while Item 10 (“Impact on future employment in your field”) measures the affective concern about that same anticipated transformation. Although the two items belong to different latent factors—one capturing perceived impact, the other capturing concern—they share a specific conceptual referent: the consequences of AI for the respondent's future career. This content overlap justifies a residual covariance beyond what is explained by the two latent factors. The inclusion of this parameter was also supported by modification indices (MI = 49.17).

To evaluate whether the latent factors should be treated as independent, we compared correlated vs. uncorrelated model, with the following specification

- **Correlated model**: the latent factors were freely allowed to correlate.
- **Uncorrelated model**: the correlation between latent factors was fixed to zero.

Model comparison was performed using a chi-square difference test (ANOVA). This procedure allowed us to determine whether the inclusion of a correlation between the latent factors significantly improved model fit. Estimation was performed with robust maximum likelihood (MLR), which accounts for non-normality in Likert-type data.

Standard fit indices were reported, including the comparative fit index (CFI), Tucker–Lewis Index (TLI), root mean square error of approximation (RMSEA), and standardized root mean square residual (SRMR).

#### Measurement invariance testing

To ensure that comparisons across groups were valid, we conducted full measurement invariance tests for gender and disciplinary area, following the ΔCFI criterion recommended by Cheung and Rensvold (2002), using MLR estimation. For gender, metric invariance (equal loadings) yielded ΔCFI = 0.002 (< 0.01), and scalar invariance (equal loadings ± intercepts) yielded ΔCFI = 0.007 (< 0.01). For disciplinary area (dichotomized as STEM vs. non-STEM), metric invariance yielded ΔCFI = 0.003 (< 0.01), and scalar invariance yielded ΔCFI = 0.007 (< 0.01). Full scalar invariance across both grouping variables indicates that the items work equivalently across groups—the factor loadings and item intercepts are statistically equivalent. This means that observed differences in latent factor scores between groups are not attributable to measurement artifacts, and that comparisons of regression coefficients and latent means across groups are fully justified. Detailed results are reported in Supplementary Table S1.

### CFA with covariates

The final model was a confirmatory factor analysis (CFA) in which latent factors were regressed on age, gender, disciplinary field, and self-reported use of AI. Gender was dummy-coded (0 = male, 1 = female). Disciplinary field was represented by four categories: STEM, Health/Agricultural/Veterinary Sciences, Economics/Law/Social Sciences, with Arts/Humanities/Education as the reference category. AI use was coded into two dummy variables: “No, never” and “Yes, occasionally,” with “Yes, regularly” as the reference category.

Models were estimated on students enrolled in bachelor's, master's, or single-cycle master's degree programs, as well as doctoral students (*N* = 864). Results are presented as standardized path coefficients to facilitate comparability.

All analyses were conducted in R (version 4.5.0; R Core Team, 2025) using the packages psych (Revelle and Revelle, 2015), lavaan (Rosseel, 2012), and semPlot (Epskamp, 2015). The full R code is reported in Supplementary material.

## Results

### Demographics

A total of 864 students participated in the survey. The mean age was 24.8 years (range: 18–56 years), with a balanced gender distribution (Table 1). The sample included students from various academic levels, predominantly 3-year degree programs (49.3%), followed by master's degree (20.5%), single-cycle master's degree (20.3%), and PhD/doctoral programs (9.7%). Students represented diverse academic areas, with Science, Technology, Engineering and Mathematics (STEM) comprising 31.3% of respondents, followed by Medicine and Life Sciences (26.2%), Economics and Law (22.3%), and Arts, Literature and Educational Sciences (20.2%). Most respondents (59.8%) were in their first or second year of study.

**Table 1:** Demographic characteristics of student participants (*N* = 864).

Characteristic	Category	*n* (%)
Gender	Male	424 (49.1)
	Female	417 (48.3)
	Other/prefer not to specify	23 (2.6)
Degree type	Three-year degree	426 (49.3)
	Master's degree	177 (20.5)
	Single-cycle Master's degree	175 (20.3)
	PhD/doctoral degree	84 (9.7)
	Old system degree	2 (0.2)
Year of study	First year	276 (31.9)
	Second year	241 (27.9)
	Third year	174 (20.1)
	Fourth year	35 (4.0)
	Fifth year	28 (3.3)
	Sixth year	11 (1.3)
	Beyond normal duration	99 (11.4)
Academic area	Science, technology, engineering and mathematics	270 (31.3)
	Medicine and life sciences	226 (26.2)
	Economics and law	193 (22.3)
	Arts, literature, and educational sciences	175 (20.2)

### AI knowledge and current usage

Regarding AI familiarity, 65.8% of students reported superficial knowledge, while 34.2% claimed good knowledge of AI technologies. Notably, no students reported having no knowledge of AI. In terms of usage patterns, 50.4% used AI applications regularly, 42.6% used them occasionally, and only 6.4% had never used AI applications. ChatGPT emerged as the most popular AI application, used by 59.8% of respondents, followed by Gemini (14.6%), Copilot (7.3%), and other tools with lower usage rates.

### Factor structure of student attitudes

#### Descriptive analysis

**Figure 1:**
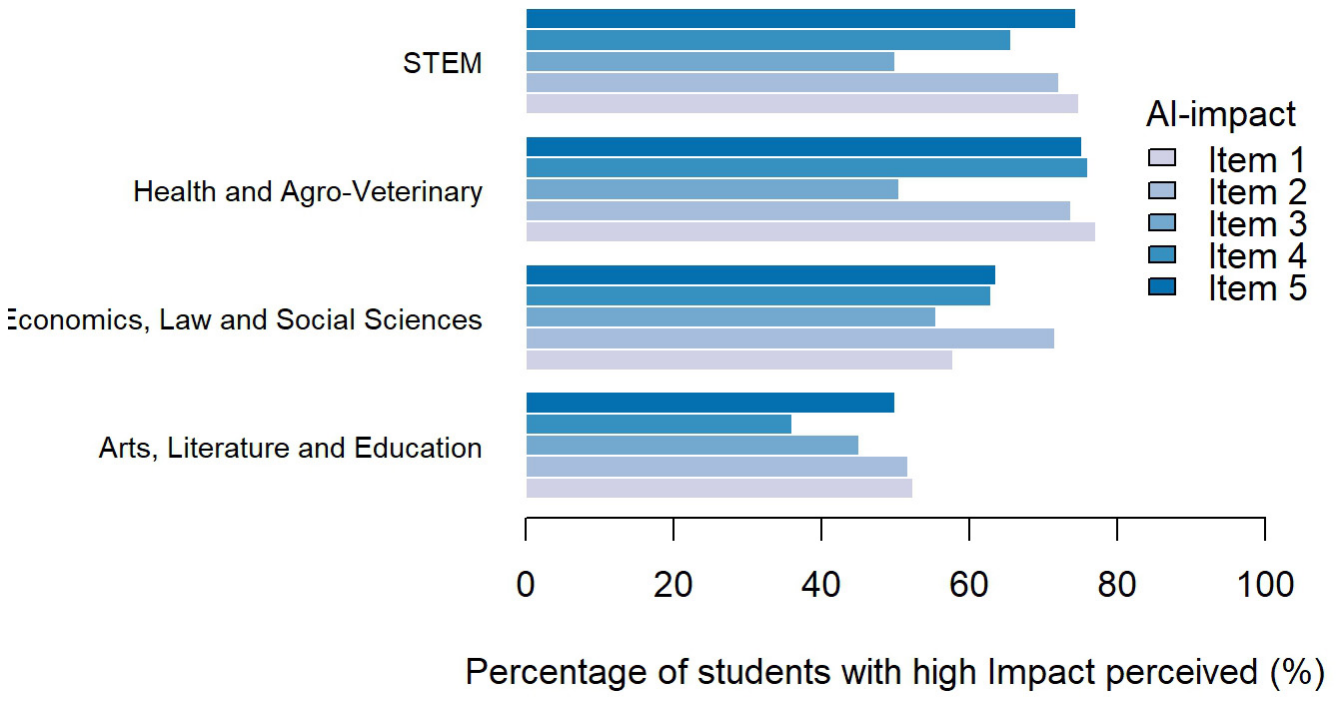
Percentage of students reporting positive perceptions of AI impact by disciplinary field. Bar plot showing the proportion of students who selected high scores (4–5) on five questions measuring AI impact perceptions (item 1–item 5), categorized across four disciplinary areas.

**Figure 2:**
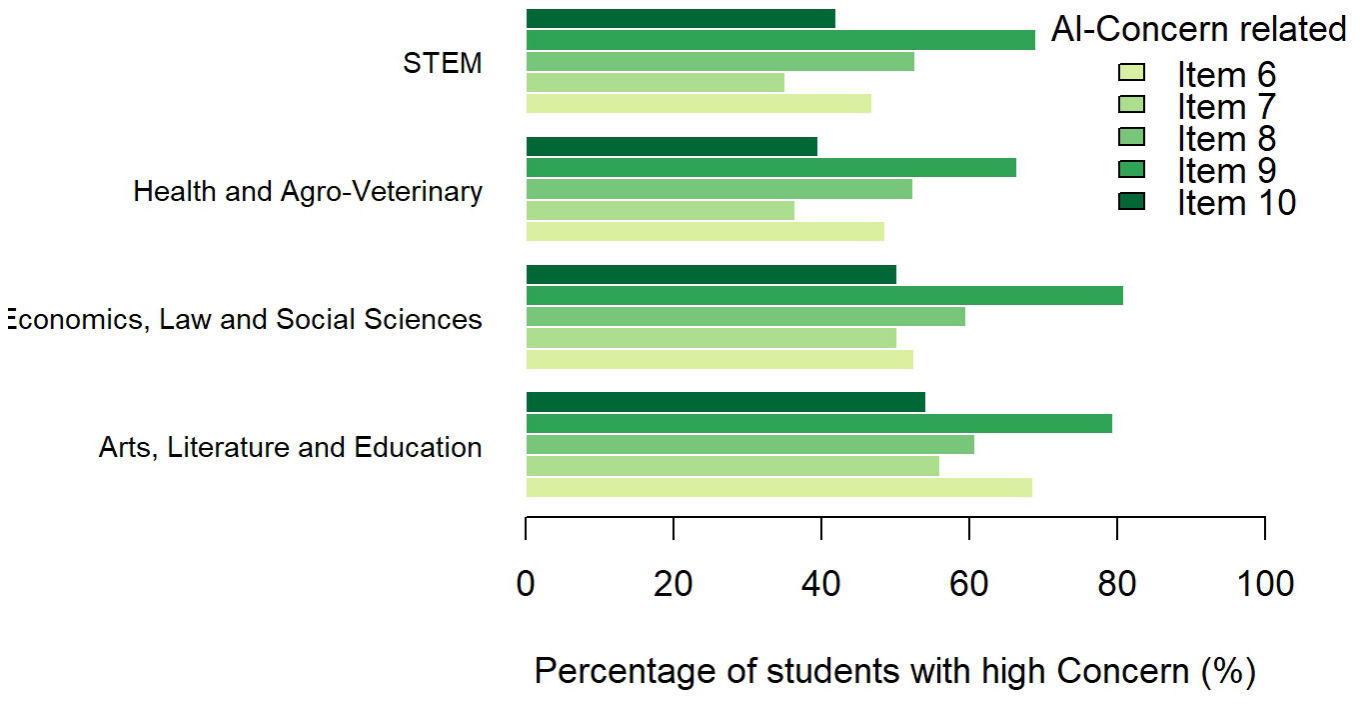
Percentage of students reporting high levels of concern about AI by disciplinary field. Bar plot showing the proportion of students who selected high scores (4–5) on five questions measuring AI-related concerns (item 6–item 10), categorized across four disciplinary areas.

The proportion of students reporting positive perceptions of AI impact or high levels of concern about AI, stratified by disciplinary field, is reported in Figures 1, 2, respectively. The students from Health and Agro-Veterinary and STEM fields show a greater propensity to select high scores (4–5) across impact questions 1–5, indicating stronger positive perceptions of AI's role in their academic and professional contexts. Students from Arts, Literature and Education and Economics, Law and Social Sciences disciplines demonstrate a greater tendency to select high concern scores (4–5) across questions 6–10, suggesting more apprehension about AI implications in these fields.

#### Correlation analysis

**Figure 3:**
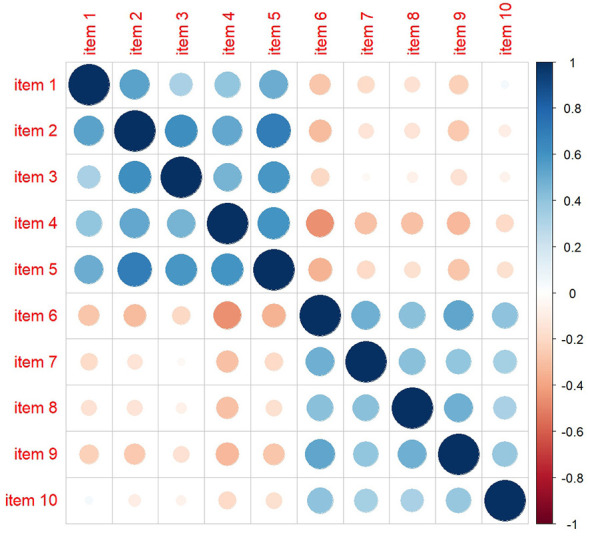
Correlation matrix between survey items. Pairwise correlation plot showing the relationships between the 10 Likert-type items measuring student attitudes toward AI. Items 1–5 (measuring Perceived Impact of AI) and Items 6–10 (measuring AI-related Concerns) exhibit positive correlations within each block and negative correlations between blocks, providing initial evidence for two distinct latent constructs. Circle size and color intensity indicate correlation strength (blue = positive correlations; red = negative correlations).

The pairwise correlation plot between items (Figure 3) reveals two distinct question blocks: items 1 to 5 and items 6 to 10 exhibit positive correlations within each block, while negative correlations are observed between the two blocks. This pattern provides initial evidence for the existence of two variable clusters, potentially measuring two distinct latent constructs related to perceived opportunities (items 1–5) and concerns (items 6–10) regarding AI integration.

#### Parallel analysis

**Figure 4:**
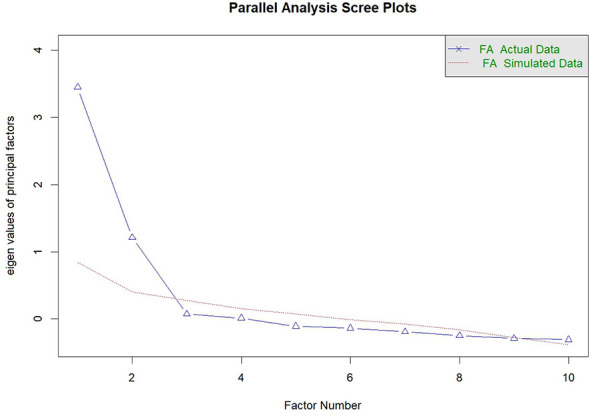
Parallel analysis scree plot. Scree plot comparing eigenvalues from the actual data (FA actual data, solid line with triangles) to eigenvalues from simulated random data (FA simulated data, dotted line). The plot indicates a clear elbow after the second factor, suggesting that a two-factor solution optimally explains the underlying structure of student attitudes toward AI integration.

The parallel analysis scree plot (Figure 4) indicates a clear elbow after the second factor, where the eigenvalues of the actual data (FA Actual Data) exceed those of the simulated data (FA Simulated Data) only for the first two factors. This suggests that a two-factor solution is optimal for explaining the underlying structure of the data.

#### Exploratory factor analysis

An exploratory factor analysis (EFA) was conducted on the 10 items using maximum likelihood extraction and oblique rotation. As suggested by the scree plot, two factors emerged with eigenvalues greater than 1, accounting together for 49% of the total variance (Factor 1: 27%; Factor 2: 23%; Table 2).

**Table 2:** Exploratory factor analysis: factor loadings and communalities.

Item	ML1 (impact)	ML2 (concern)	Communality (h^2^)	Uniqueness (u^2^)	Complexity
1	0.57	−0.05	0.35	0.65	1.0
2	0.85	0.03	0.71	0.29	1.0
3	0.75	0.12	0.51	0.49	1.0
4	0.56	−0.27	0.51	0.49	1.4
5	0.82	−0.05	0.71	0.29	1.0
6	−0.13	0.69	0.56	0.44	1.1
7	0.06	0.66	0.41	0.59	1.0
8	0.06	0.66	0.41	0.59	1.0
9	−0.06	0.67	0.48	0.52	1.0
10	0.09	0.57	0.29	0.71	1.0
SS loadings	2.68	2.25			
Proportion var	0.27	0.23			
Cumulative var	0.27	0.49			

ML1 = factor 1 (perceived impact of AI); ML2 = factor 2 (AI-related concerns). h^2^ = communality; u^2^ = uniqueness. Extraction: maximum likelihood; rotation: oblimin.

The pattern of factor loadings indicated a clear two-factor solution. Items 1–5 loaded primarily on Factor 1 (ML1), with standardized loadings ranging from 0.56 to 0.85, while Items 6–10 loaded primarily on Factor 2 (ML2), with loadings ranging from 0.57 to 0.69. All communalities were acceptable (*h*^2^ = 0.29–0.71), suggesting that the items were adequately represented by the two extracted factors. Cross-loadings were minimal, with only Item 4 showing some secondary loading (complexity = 1.4), but not to an extent that compromised the interpretation of the factors. Based on item content, Factor 1 was labeled “Perceived Impact of AI” and Factor 2 was labeled “AI-related Concerns.”

#### Reliability

The perceived impact factor demonstrated good internal consistency [Cronbach's α = 0.85; 95% CI (0.83–0.86); McDonald's ω = 0.85]. The AI-related Concerns factor demonstrated acceptable internal consistency [Cronbach's α = 0.78; 95% CI (0.76–0.80); McDonald's ω = 0.78].

### Confirmatory factor analysis

To test the two-factor structure identified in the exploratory factor analysis, a confirmatory factor analysis (CFA) was conducted. The model included two latent constructs: perceived impact of AI (loaded by Items 1–5) and AI-related Concerns (loaded by Items 6–10).

To evaluate whether the two factors should be treated as independent, we compared the correlated-factors model to an alternative model in which the correlation between the two factors was constrained to zero. The robust Satorra–Bentler scaled chi-squared difference test indicated that the uncorrelated model fit the data significantly worse than the correlated model (Δχ^2^ = 90.60, Δdf = 1, *p* < 0.001). Based on this comparison, the correlated-factors model was retained.

The final CFA model—including covariates and the residual covariance between Item 1 and Item 10—demonstrated acceptable fit to the data: $\chi_{\left(89\right)}^{2}$ = 414.58, *p* < 0.001; robust CFI = 0.905; robust TLI = 0.878; RMSEA = 0.067 [90% CI (0.061, 0.073)]; SRMR = 0.047. We acknowledge that the TLI value (0.878) falls marginally below the conventional threshold of 0.90. However, two factors contribute to this: (a) model complexity—the model includes 10 indicators, two latent factors, and seven observed covariates, and the TLI penalizes model complexity more heavily than the CFI (Marsh et al., 2004); and (b) sample size sensitivity—with *N* = 864, the chi-square statistic is inflated, which tends to depress the TLI (Hu and Bentler, 1999). Given that CFI = 0.905, RMSEA = 0.067, and SRMR = 0.047 all meet conventional thresholds, we consider the overall model fit adequate.

All items loaded significantly on their respective latent factors (*p* < 0.001; Table 3). Standardized loadings ranged from 0.59 to 0.85 for Perceived Impact of AI and from 0.53 to 0.75 for AI-related Concerns, supporting good internal consistency and convergent validity. Finally, the two latent factors were significantly and negatively correlated (*r* = −0.45, *p* < 0.001), suggesting that higher perceived impact was associated with lower levels of concern.

**Table 3:** Confirmatory factor analysis: standardized factor loadings.

Item	Estimate	95% CI	p-value	Std. All
Factor 1: perceived impact of AI				
Item 1	1.00	—	—	0.59
Item 2	1.53	[1.35, 1.71]	< 0.001	0.83
Item 3	1.42	[1.22, 1.62]	< 0.001	0.69
Item 4	1.00	[0.80, 1.20]	< 0.001	0.68
Item 5	1.68	[1.46, 1.90]	< 0.001	0.85
Factor 2: AI-related CONCERNS				
Item 6	1.00	—	—	0.75
Item 7	0.83	[0.73, 0.93]	< 0.001	0.61
Item 8	0.84	[0.72, 0.96]	< 0.001	0.62
Item 9	0.89	[0.79, 0.99]	< 0.001	0.71
Item 10	0.72	[0.62, 0.82]	< 0.001	0.53
Factor correlation				
Impact ~ concern	−0.267	[−0.33, −0.21]	< 0.001	−0.45

95% CI, 95% confidence interval computed as estimate ± 1.96 × SE. Std. All = standardized solution. Item 1 (factor 1) and item 6 (factor 2) are reference indicators (loading fixed to 1.00; CI not applicable). Estimator: MLR. Residual covariance estimated between Item 1 and Item 10.

Predictors showed distinct patterns of association with the two latent factors (Table 4).

**Table 4:** Regression Coefficients on the Latent Factors Identified by CFA.

Latent factor	Perceived impact of AI				AI-related concerns			
	Estimate	95% CI	*p*-value	Sth. β	Estimate	95% CI	*p*-value	Std. β
Age	0.014	[0.01, 0.02]	< 0.001	**0.17**	−0.005	[−0.02, 0.01]	0.411	–**0.04**
Gender (female)	−0.109	[−0.20, −0.02]	0.013	–**0.09**	0.402	[0.26, 0.54]	< 0.001	**0.21**
Field of study								
STEM	0.261	[0.12, 0.40]	< 0.001	**0.19**	−0.221	[−0.42, −0.02]	0.033	–**0.11**
Health/Agr/Vet	0.314	[0.18, 0.45]	< 0.001	**0.23**	−0.300	[−0.51, −0.09]	0.004	–**0.15**
Ec/legal/social	0.243	[0.11, 0.38]	< 0.001	**0.16**	−0.068	[–−0.28, 0.15]	0.531	–**0.03**
AI use								
No, never	−0.928	[−1.16, −0.70]	< 0.001	—-**0.35**	0.818	[0.55, 1.08]	< 0.001	**0.20**
Yes, occasionally	−0.441	[−0.54, −0.35]	< 0.001	–**0.35**	0.542	[0.39, 0.69]	< 0.001	**0.29**
Model fit								
*R* ^2^				**0.72**				**0.79**

95% CI, 95% confidence interval computed as estimate ± 1.96 × SE. Std. β = standardized path coefficient. Reference categories: humanities/arts (field of study); frequent AI use (AI Use). Estimator: MLR. *R*^2^ values reflect variance explained in each latent factor. bold values indicate standardized path coefficients.

Perceived Impact was positively predicted by age (β = 0.17, *p* < 0.001), STEM field (β = 0.19, *p* < 0.001), Health/Agricultural/Veterinary sciences (β = 0.23, *p* < 0.001), and Economics/Law/Social Sciences (β = 0.16, *p* < 0.001). Conversely, it was negatively predicted by female gender (β = −0.09, p =0.013), “never user” status (β = −0.35, *p* < 0.001), and “occasional user” status (β = −0.35, *p* < 0.001).

AI-related Concern was positively predicted by female gender (β = 0.21, *p* < 0.001), “never user” status (β = 0.20, *p* < 0.001), and “occasional user” status (β = 0.29, *p* < 0.001), while it was negatively predicted by STEM field (β = −0.11, *p* = 0.033) and Health/Agricultural/Veterinary disciplines (β = −0.15, *p* = 0.004). Age and Economics/Law/Social Sciences were not significant predictors of concern.

The partial association between the two latent constructs after accounting for the covariates (age, gender, disciplinary area, and AI use frequency) was negative and statistically significant (*r* = −0.343, *p* < 0.001). This value represents the residual correlation between Perceived Impact and AI-related Concerns after controlling for the covariates and suggests that higher perceived impact was associated with lower concern.

The model demonstrated substantial explanatory power, with *R*^2^ = 0.72 for Perceived Impact and *R*^2^ = 0.79 for AI-related Concerns.

## Discussion

This comprehensive survey of students at the University of Palermo (Italy) reveals a multidimensional landscape of AI perceptions within the Italian higher education context, providing important insights into how students organize their attitudes toward AI integration and which factors systematically predict these attitudes. The findings demonstrate that student perceptions are not unidimensional but rather reflect two distinct yet related psychological constructs: recognition of AI's transformative potential and concern about its risks.

Beyond the educational domain, research conducted in Middle Eastern organizational contexts has examined how contextual variables—including institutional readiness, trust, and perceived usefulness—shape individual and organizational responses to AI adoption (Abdulmuhsin et al., 2025; Alkhwaldi et al., 2026). These findings reinforce the relevance of contextual and psychological moderators in AI adoption, a pattern that parallels the disciplinary and experiential predictors identified in the present study.

In our study, the factor analytic findings provide strong evidence that student attitudes toward AI in higher education are organized along two independent dimensions: *perceived impact of AI* and *AI-related concerns*. This bidimensional structure has important theoretical and practical implications. Rather than viewing students as simply “pro-AI” or “anti-AI,” our results suggest that students simultaneously hold nuanced perspectives that recognize both opportunities and risks. The moderate negative correlation between these dimensions (*r* = −0.34, after accounting for covariates) indicates that while students who perceive greater benefits tend to have fewer concerns, these constructs are distinct and can coexist.

One of the most robust findings from the confirmatory factor analysis is the powerful effect of AI usage patterns on both attitude dimensions. Students who regularly use AI applications showed significantly higher perceived impact and lower concerns compared to occasional users and non-users. These large effect sizes suggest that direct, hands-on experience with AI tools is among the strongest predictors of positive attitudes and reduced anxiety. This pattern aligns with established technology adoption literature, including the Technology Acceptance Model proposed by Davis (1989), showing that direct experience reduces uncertainty and increases perceived usefulness (Bonfield et al., 2020; Ertmer and Ottenbreit-Leftwich, 2010)). The superficial nature of most students' AI knowledge (65.8%) despite high usage rates (93.6%) suggests that current engagement with AI tools is largely informal and self-directed rather than pedagogically structured. Students appear to be learning about AI primarily through experimentation with applications like ChatGPT rather than through formal instruction that provides deeper understanding of AI capabilities, limitations, and appropriate use contexts. As noted by Kelly and Sullivan (2023), generative AI tools like ChatGPT offer significant opportunities for personalized learning and research acceleration, yet they simultaneously raise concerns about academic integrity and the fundamental nature of human learning. Similarly, Rudolph et al. (2023) argue that these technologies challenge traditional assessment practices, requiring educators to reconsider what constitutes authentic student work. The implication of this finding is that universities should provide students with structured, supervised opportunities to engage with AI tools in educational contexts. Mere exposure to AI concepts through lectures is unlikely to be as effective as active, hands-on learning experiences that allow students to directly observe AI's benefits and limitations in their own academic work.

The analysis revealed significant gender differences in both attitude dimensions. Female students showed lower perceived impact and higher AI-related concerns compared to male students. While the effect on perceived impact was modest (β = −0.09), the effect on concerns was among the larger coefficients in the model (β = 0.21), suggesting that female students experience significantly greater anxiety about AI's potential negative consequences. We interpret these findings through three non-mutually exclusive explanations. First, lower prior exposure and self-efficacy: female students consistently report lower perceived knowledge of AI and lower exposure awareness compared to male peers (Cachero et al., 2025). This reflects longstanding gendered socialization patterns that discourage women from engaging with technical domains, leading to lower technology self-efficacy independently of actual proficiency (Matobobo, 2026). Second, higher sensitivity to ethical risks: women may hold a more realistic appraisal of AI-specific risks, including documented tendencies of AI hiring tools to reproduce gender biases and disproportionate labor market vulnerability (Huseynov, 2025). Empirical studies confirm that female students report higher AI anxiety and more negative attitudes toward AI (Russo et al., 2025). Third, differential technology socialization: broader cultural norms linking AI and technology with masculinity lead women to adopt a more cautious stance toward emerging technologies, independently of their actual skills (Russo et al., 2025).

Our survey found significant disciplinary differences in AI attitudes. Students in STEM, Health/Agricultural/Veterinary sciences, and Economics/Law/Social Sciences showed significantly higher perceived impact compared to Arts/Humanities/Education students. Conversely, STEM and Health/Agricultural/Veterinary students reported lower concerns. These disciplinary patterns likely reflect both differences in curriculum content and professional culture. STEM and health science students are more likely to encounter AI applications in their coursework and future professions, making AI's relevance more immediately apparent (Long and Magerko, 2020). The fact that Arts/Humanities/Education students showed the lowest perceived impact and highest concerns is particularly noteworthy. These disciplines play crucial roles in developing critical thinking, ethical reasoning, and human-centered perspectives that are essential for responsible AI development and deployment. We propose two complementary explanations. First, fear of dehumanization: humanities students may perceive AI as a direct threat to disciplines whose core tasks—textual analysis, creative writing, interpretive work—AI systems can now perform. Non-STEM students may update their beliefs about future earnings more pessimistically in response to AI-related information, suggesting a greater sense of occupational vulnerability. Reiter et al. (2025) confirm that humanities students consistently report more negative attitudes and greater concerns about AI than their STEM counterparts. Second, differences in digital literacy training: STEM curricula systematically develop computational thinking and data literacy, fostering familiarity and agency with algorithmic systems, while Humanities curricula typically lack this foundational training (Tariq et al., 2025). The HEPI/Kortext Survey (Freeman, 2025) documents that Arts and Humanities students have substantially less prior experience with AI tools, consistent with this interpretation.

The positive relationship between age and perceived impact, while modest, suggests that older students recognize AI's significance more strongly than younger peers. This finding is somewhat counterintuitive given common assumptions about younger “digital natives” being more technologically enthusiastic. However, older students may have more developed professional perspectives that help them appreciate AI's practical implications for their careers or may have accumulated more diverse experiences with technology adoption that provide context for understanding AI's transformative potential.

The findings from this study have several important implications for universities seeking to integrate AI into their curricula. Given the powerful effect of direct AI experience on attitudes, universities should prioritize providing students with supervised opportunities to engage with AI tools in educational contexts. These programs should move beyond superficial tool usage to develop deeper understanding of AI capabilities, limitations, and appropriate applications (Long and Magerko, 2020). The disciplinary divide in perceived impact suggests that generic AI training may not resonate equally across fields. Humanities and social science students need to see AI's relevance to their disciplines through concrete, field-specific applications that align with their professional goals and values. The bidimensional structure of attitudes indicates that addressing concerns does not automatically increase perceived benefits. Successful implementation requires parallel strategies: highlighting AI's educational potential while simultaneously providing concrete solutions to legitimate concerns about privacy, employment, and human interaction. Finally, given the strong effect of AI usage on attitudes, creating communities where students can share experiences, troubleshoot problems, and learn from peers who have successfully integrated AI into their academic work may be more effective than top-down training programs.

Several important limitations must be considered when interpreting these findings. First, the response rate of 1.88% and the self-selected nature of participation may introduce selection bias toward students with pre-existing interest in AI. Second, the single-institution design limits generalizability to other contexts. Third, the rapid evolution of AI technologies implies that perceptions may change over time. Fourth, the 10 items were developed *de novo* without prior pilot testing; therefore, the factor structure should be considered preliminary and requires validation in future research with refined instruments. Finally, prior research shows that female students use AI tools less frequently than male students. The gender effects reported here may therefore be partially confounded with differential AI use frequency. While formally testing a mediation model falls outside the scope of the present study, future research should formally test whether AI use frequency mediates the relationship between gender and AI attitudes.

Future research should address these limitations through longitudinal studies examining how attitudes evolve over time and in response to specific interventions. Comparative research across different institutional contexts would help identify universal principles for AI integration while accounting for local variations. Investigation of actual learning outcomes associated with AI-enhanced education would provide crucial evidence for evidence-based decision making (Zawacki-Richter et al., 2019; Kelly and Sullivan, 2023). Qualitative research exploring specific concerns and opportunities identified by different student subgroups could provide richer understanding of the psychological processes underlying the quantitative patterns observed here. Finally, experimental studies manipulating specific aspects of AI literacy interventions could identify which pedagogical approaches most effectively shape student attitudes and competencies (Tlili et al., 2023).

In conclusion, this study reveals that student attitudes toward AI in higher education are organized into two distinct but related dimensions reflecting opportunity recognition and risk awareness. These perceptions are systematically influenced by direct AI experience, gender, academic discipline, and age. The strong effect of AI usage on both perceived impact and concerns suggests that providing students with structured, hands-on experiences with AI tools in educational contexts should be a central priority for universities seeking to foster positive attitudes and reduce anxiety. Successful AI implementation requires tailored strategies that address discipline-specific relevance, and recognize both the opportunities and risks that AI presents for higher education.

## Data Availability

The raw data supporting the conclusions of this article will be made available by the authors, without undue reservation.
