# Supplementary material for: Student perceptions of artificial intelligence in higher education: a structural analysis at an Italian university
Source: Front Psychol. 2026 Jun 29;17:1861001. doi: 10.3389/fpsyg.2026.1861001 (PMC13357671; doi:10.3389/fpsyg.2026.1861001)
Supplement: Supplementary file 1 [file Supplementary_file_1.docx]

**Supplementary Materials**

**Survey Items: Student Attitudes Toward AI Integration in Higher Education**

**Instructions**: Please indicate your level of agreement/concern for each statement using a 5-point Likert scale.

**Factor 1: Perceived Impact of AI (Items 1-5)**

**Scale**: 1 = Strongly Disagree, 2 = Disagree, 3 = Neutral, 4 = Agree, 5 = Strongly Agree

**Item 1**. AI will have a significant impact on my future professional field.

**Item 2**. AI can improve the quality of university education.

**Item 3**. The introduction of a basic AI course should be mandatory for all degree programs.

**Item 4**. I consider it important to acquire basic AI competencies during my university studies.

**Item 5**. I am interested in deepening my knowledge of AI applications.

**Factor 2: AI-related Concerns (Items 6-10)**

**Scale**: 1 = Not at all concerned, 2 = Slightly concerned, 3 = Moderately concerned, 4 = Very concerned, 5 = Extremely concerned

**Item 6**. Please indicate your level of concern about the possible reduction of human interaction in learning.

**Item 7**. Please indicate your level of concern about the potential increase in the digital divide among students.

**Item 8**. Please indicate your level of concern about privacy and data security risks.

**Item 9**. Please indicate your level of concern about excessive technological dependency.

**Item 10**. Please indicate your level of concern about the impact on future employment in your field.

**Table S1**. Results of Measurement Invariance Tests Across Gender and Disciplinary Area

| **Covariate** | **Comparison** | **CFI Configural** | **CFI Metric** | **CFI Scalar** | **ΔCFI (C→M)** | **ΔCFI (M→S)** |
| --- | --- | --- | --- | --- | --- | --- |
| Gender | Configural → Metric | .934 | .932 | — | .002 | — |
|  | Metric → Scalar | — | .932 | .926 | — | .007 |
| Stem vs Non Stem | Configural → Metric | .934 | .931 | — | .003 | — |
|  | Metric → Scalar | — | .931 | .925 | — | .007 |

**R Code**

The following R code reproduces all psychometric analyses reported in the main text and supplementary tables, including the EFA, CFA, reliability estimates, measurement invariance tests, and the CFA with covariates.

library(psych)

library(lavaan)

library(semPlot)

library(corrplot)

# ── EXPLORATORY FACTOR ANALYSIS (EFA) ─────────────────────────────────────────

# Correlation matrix

cor_matrix <- cor(items_efa)

corrplot(cor_matrix, method = "circle")

# Parallel analysis

fa.parallel(items_efa, fa = "fa")

# EFA with ML extraction and oblimin rotation

efa_oblimin <- fa(items_efa, nfactors = 2, rotate = "oblimin", fm = "ml")

print(efa_oblimin)

# ── CONFIRMATORY FACTOR ANALYSIS (CFA) ────────────────────────────────────────

cfa_model <- '

impact =~ item1 + item2 + item3 + item4 + item5

concern =~ item6 + item7 + item8 + item9 + item10

impact ~~ concern

item1 ~~ item10

'

cfa_uncorrelated <- '

impact =~ item1 + item2 + item3 + item4 + item5

concern =~ item6 + item7 + item8 + item9 + item10

impact ~~ 0*concern

item1 ~~ item10

'

fit_correlated <- cfa(cfa_model, data = items_efa, estimator = "MLR")

fit_uncorrelated <- cfa(cfa_uncorrelated, data = items_efa, estimator = "MLR")

# Chi-square difference test

anova(fit_correlated, fit_uncorrelated)

# Model fit summary

summary(fit_correlated, fit.measures = TRUE, standardized = TRUE)

# ── RELIABILITY: CRONBACH'S ALPHA & McDONALD'S OMEGA ─────────────────────────

# Impact factor (item1–item5)

alpha_impact <- psych::alpha(items_efa[, c("item1","item2","item3","item4","item5")])

omega_impact <- psych::omega(items_efa[, c("item1","item2","item3","item4","item5")], nfactors = 1)

# Concern factor (item6–item10)

alpha_concern <- psych::alpha(items_efa[, c("item6","item7","item8","item9","item10")])

omega_concern <- psych::omega(items_efa[, c("item6","item7","item8","item9","item10")], nfactors = 1)

print(alpha_impact); print(alpha_concern)

cat("Omega impact: ", round(omega_impact$omega.tot, 3), "\n")

cat("Omega concern:", round(omega_concern$omega.tot, 3), "\n")

# ── MEASUREMENT INVARIANCE TESTING ────────────────────────────────────────────

invariance_model <- '

impact =~ item1 + item2 + item3 + item4 + item5

concern =~ item6 + item7 + item8 + item9 + item10

impact ~~ concern

item1 ~~ item10

'

dati_stud$stem_vs_nonstem <- ifelse(

dati_stud$area_disciplinare == "STEM", "STEM", "non-STEM"

)

# Gender

fit_configural_gender <- cfa(invariance_model, data = dati_stud,

group = "gender_female", estimator = "MLR", meanstructure = TRUE)

fit_metric_gender <- cfa(invariance_model, data = dati_stud,

group = "gender_female", group.equal = "loadings",

estimator = "MLR", meanstructure = TRUE)

fit_scalar_gender <- cfa(invariance_model, data = dati_stud,

group = "gender_female", group.equal = c("loadings","intercepts"),

estimator = "MLR", meanstructure = TRUE)

# Disciplinary area (STEM vs. non-STEM)

fit_configural_disc <- cfa(invariance_model, data = dati_stud,

group = "stem_vs_nonstem", estimator = "MLR", meanstructure = TRUE)

fit_metric_disc <- cfa(invariance_model, data = dati_stud,

group = "stem_vs_nonstem", group.equal = "loadings",

estimator = "MLR", meanstructure = TRUE)

fit_scalar_disc <- cfa(invariance_model, data = dati_stud,

group = "stem_vs_nonstem", group.equal = c("loadings","intercepts"),

estimator = "MLR", meanstructure = TRUE)

# ── CFA WITH COVARIATES ────────────────────────────────────────────────────────

# Dummy variables

dati_stud$stem_dummy <- ifelse(dati_stud$area_disciplinare == "STEM", 1, 0)

dati_stud$health_dummy <- ifelse(dati_stud$area_disciplinare == "Sanitaria e Agro-Veterinaria", 1, 0)

dati_stud$econlaw_dummy <- ifelse(dati_stud$area_disciplinare == "Economica, Giuridica e Sociale", 1, 0)

dati_stud$ai_never <- ifelse(dati_stud$utilizzo_ai == "NO, MAI", 1, 0)

dati_stud$ai_occasional <- ifelse(dati_stud$utilizzo_ai == "SI, OCCASIONALMENTE", 1, 0)

cfa_covariates_model <- '

impact =~ item1 + item2 + item3 + item4 + item5

concern =~ item6 + item7 + item8 + item9 + item10

item1 ~~ item10

impact ~ eta + gender_female + stem_dummy + health_dummy +

econlaw_dummy + ai_never + ai_occasional

concern ~ eta + gender_female + stem_dummy + health_dummy +

econlaw_dummy + ai_never + ai_occasional

'

fit_cfa_cov <- cfa(cfa_covariates_model, data = dati_stud, estimator = "MLR")

summary(fit_cfa_cov, fit.measures = TRUE, standardized = TRUE)
